# An Accessible Smart Home Based on Integrated Multimodal Interaction

**DOI:** 10.3390/s21165464

**Published:** 2021-08-13

**Authors:** Ana Patrícia Rocha, Maksym Ketsmur, Nuno Almeida, António Teixeira

**Affiliations:** Department of Electronics, Telecommunications and Informatics, Institute of Electronics and Informatics Engineering of Aveiro, University of Aveiro, 3810-193 Aveiro, Portugal; mvk@ua.pt (M.K.); nunoalmeida@ua.pt (N.A.)

**Keywords:** conversational assistants, multimodal interaction, smart homes, sensors and devices, real scenarios, accessibility

## Abstract

Our homes are becoming increasingly sensorized and smarter. However, they are also becoming increasingly complex, making accessing them and their advantages difficult. Assistants have the potential for improving the accessibility of smart homes, by providing everyone with an integrated, natural, and multimodal way of interacting with the home’s ecosystem. To demonstrate this potential and contribute to more environmentally friendly homes, in the scope of the project Smart Green Homes, a home assistant highly integrated with an ICT (Information and communications technology) home infrastructure was developed, deployed in a demonstrator, and evaluated by seventy users. The users’ global impression of our home assistant is in general positive, with 61% of the participants rating it as good or excellent overall and 51% being likely or very likely to recommend it to others. Moreover, most think that the assistant enhances interaction with the smart home’s multiple devices and is easy to use by everyone. These results show that a home assistant providing an integrated view of a smart home, through natural, multimodal, and adaptive interaction, is a suitable solution for enhancing the accessibility of smart homes and thus contributing to a better living ambient for all of their inhabitants.

## 1. Introduction

Led by pervasive computing and the Internet of Things (IoT), our homes are becoming increasingly sensorized, with the installation of a growing number of diverse smart appliances and devices (e.g., smart lights, dishwasher, and air quality sensors) with embedded sensors and actuators [1,2,3,4]. Besides enabling device control, smart homes potentially allow acquiring a great variety of relevant and valuable information on the home and its devices.

Smart homes have the great potential to improve the quality of life of their inhabitants, by enabling more comfortable, secure, healthy, independent, assisted living [1,5]. However, they are also becoming increasingly more complex, making it difficult to interact with them in a simple and easy way. Therefore, to allow everyone to take full advantage of their smart home, it is important to have a unified view of all devices that allows dealing with the complexity of the home’s ecosystem. This view can also lead to more informed decisions of the users concerning the control of their home’s devices.

Virtual assistants, such as Google Assistant [6], Amazon’s Alexa [7], and Apple’s Siri [8], are already available on different devices (e.g., smartphone, smartwatch, car, TV, and laptop) and are used by many to help them with various tasks [9]. In the context of smart homes, assistants supporting conversation capabilities are very popular, with people using for example smart speakers or displays with integrated assistants (e.g., Google Nest/Home [6], Amazon Echo [10], and Apple’s HomePod [11]) to control the different smart devices installed in their homes (e.g., lights, locks, thermostats, and TV) [3].

Assistants have the potential of providing a natural and integrated way of interacting with smart homes. However, despite their popularity and recent advancements, commercially available home assistants still have some limitations, for example, they tend not to provide a unified view on the home, i.e., they do not store data regarding the home devices in a structured way, not allowing to perform rich complex queries for obtaining relevant information (e.g., consumption of a resource by all devices in a given home division) [12]. Multi-turn conversation is also either lacking or needs to be improved to become more natural and engaging [13,14]. Moreover, home assistants typically do not support adaptation of the interaction modalities to the different home inhabitants and contexts, focusing mostly only on personalizing the content (e.g., calendar, email, and music) [15]. Also very important are the conclusions of Lopez and coworkers that show there is room for improvement when it comes to the usability of “speech-based natural user interfaces” such as virtual assistants [16].

Next generations of home assistants need to contemplate new capabilities and be more aligned with users’ needs. A recent inquiry [12], by the authors, to 20 participants aged between 10 and 63 years old, with different backgrounds, revealed some capabilities deemed important by users: home state report, temperature control of appliances (e.g., water heater, oven), and information regarding resources consumption.

Smart home’s devices also include mobile devices, such as laptops, tablets, and smartphones, which are already commonly used on a daily basis by most people. These devices are usually equipped with various sensors (e.g., touch screen, camera, microphone, and speaker) and can therefore be used as an alternative to dedicated smart speakers/displays to enable natural multimodal interaction. The same sensors can be used to adapt the interaction to not only the user but also the context.

Nowadays, much attention has been paid to the development of Ambient Assisted Living solutions. However, there seems to be no clear support of accessibility for all, with most systems targeting a specific group of people with needs, such as older people and/or people with certain disabilities. The users of a smart home can be very diverse, possibly including children, and both younger and older adults, who have different characteristics, capabilities, and preferences [1,17]. For this reason, it is essential to provide smart home solutions that are more inclusive and accessible to everyone, by using a design and development for all.

The main aim of our work is to enhance living for all in smart homes by adopting an integrated, natural, and accessible way of interacting with the home. In this contribution, we evaluate the potential of using a home assistant to simplify the interaction between humans and the complex ecosystems of smart homes. Tests with seventy end users were carried out based on the demonstration of a person using our home assistant to obtain information and control different appliances/devices deployed at a space built specifically for the Smart Green Homes project.

In general, the opinion regarding our assistant is positive, with 61% of the participants rating it as good or excellent overall. Half would likely or very likely recommend it to others and 63% would be interested or very interested in using the assistant (and 72% of them would pay for it). Most participants considered that the assistant enhances interaction with the smart home’s multiple devices (especially with devices that are difficult to access or do not have an interface—81% and 84%, respectively) and that it is easy to use by everyone. Automatic alerts in abnormal or potentially dangerous situations stood out as the most useful assistant’s capability (chosen by 82% of the participants). The assistant was also considered to be highly desirable, being mostly associated with positive words/multi-word expressions, such as useful, easy to use, accessible, integrated, intuitive, time-saving, and friendly.

These results support the idea that assistants can be very useful for providing an integrated multimodal interaction with smart homes, not only allowing tackling their complexity, but also enhancing their inclusiveness and accessibility.

### 1.1. Smart Green Homes Project

This work was carried out in the scope of the “Smart Green Homes” project [18], which is a joint project between the University of Aveiro and Bosch Termotecnologia Aveiro. The main objective of this project is to develop integrated solutions of products and technologies for home environments, which improve comfort, security, and usability, while also contributing to greater energy efficiency. They include a solution for interaction with the smart home to obtain relevant information on its diverse devices and associated resources, as well as to control the devices.

### 1.2. Contributions

The main contributions of our work are presented below.

- Home Assistant—Design and implementation of a prototype of a home assistant characterized by (1) the adoption of an integrated view of the home; (2) redundant (multimodal) interaction, both for input and output; (3) user and context adaptation capabilities; (4) multi-turn conversation; and (5) user and system initiative.
- Evolution of AM4I Architecture—Addition of support for adaptation to the user and context to the state-of-the-art interaction architecture AM4I (Adaptive Multiplatform Multidevice Multilingual Multimodal Interaction) [19], and the demonstration of the AM4I capabilities for smart home interaction.
- Demonstrator—Integration of the implemented home assistant in a rich real scenario, with real devices.
- End Users Perception—New insights on conversational assistants, resulting from a study with a large group of end users.
- Increased Accessibility—End users’ opinions highlight the usability, usefulness, ease-of-use and accessibility of the proposed solution.

### 1.3. Paper Structure

The remainder of this paper is organized as follows. The next section presents some background and related work regarding smart homes’ appliances and devices, and interaction with smart homes. In Section 3, we introduce the demonstrator developed for evaluating our assistant in a real setting, including the considered scenario, the demonstrator’s architecture, and some implementation details. The assistant is presented in Section 4, including the defined requirements, used development approach, architecture, and details on the home’s information structuring and adaptive interaction. Section 5 describes the assistant’s evaluation by end users. The obtained results are reported in Section 6 and then discussed in Section 7. Finally, the main conclusions of our work are laid out in Section 8, which also includes possible future research directions.

## 2. Background and Related Work

This section presents background information and related work on topics relevant to the present contribution, namely, smart homes’ appliances and devices, which include sensors and actuators, and interaction with smart homes involving home assistants, and multimodal and/or adaptive interaction.

### 2.1. Smart Homes and Their Appliances, Devices, Sensors, and Actuators

A smart home is “a residence equipped with a communications network, linking sensors, domestic appliances, and devices, that can be remotely monitored, accessed or controlled, and which provides services that respond to the needs of its inhabitants” [20].

Recent years have seen an increase in the number of smart appliances and devices available for installation in our homes [1,4,5], which often have various embedded sensors and/or actuators. Sensors measure physical input from their environment and convert it to data that can be interpreted by either a human or a machine, while actuators convert instructions into a mechanical action.

Examples of smart appliances or devices, as well as information that can be obtained by the sensors and/or control actions that can be performed by the actuators, are the following [1,21,22,23]:

- Smart lights, plugs, and locks—obtain and control their state (e.g., on/off and intensity in the case of the lights);
- Motion detectors/sensors—measure motion to detect the presence of people;
- Thermostats—measure and adjust the home temperature;
- Air quality sensors—measure the indoor air quality;
- Kitchen appliances (e.g., refrigerator, dishwasher)—obtain information and control them (e.g., malfunction or leak detection, door state, and temperature adjustment for the fridge, start and check the status of a washing cycle for the dishwasher).

### 2.2. Interaction with Smart Homes

Although smart homes enable greater automation, the autonomy of their users should be preserved by giving them full control of their home, instead of the home autonomously deciding on what is best for them [1,17,24]. For example, users should be able to program themselves automated tasks, such as turning on/off a device at specific times, and decide when to (de)active a given feature of a device, such as activating the air purification after being warned about the air quality reaching a certain level.

For users to be in control of their homes, it is necessary to provide a way of interacting with them, allowing access to relevant information and control of the different devices. There are several input and output modalities that can be used for interaction, including text, speech, graphical, touch, gestures, and gaze [19].

In the context of smart homes, there are available solutions based on graphical and/or touch modalities, with most of them being proprietary solutions that allow the configuration and control of only specific devices (e.g., Philips’ Hue apps for their smart lights [25] and the Kasa Smart app for TP-Link smart devices [26]). However, the most popular way of interacting with smart homes nowadays is through speech using voice assistants with conversational capabilities, which allow consulting information and control devices from multiple brands [27,28].

These assistants are commonly integrated into smart speakers or displays [6,10,11]. Although smart speakers allow for more natural interaction, this interaction is limited to speech input and output. Smart displays provide additional forms of interaction, such as touch, text, and graphical modalities. However, similarly to speakers, they are typically installed at a specific home location (e.g., bedroom, kitchen, or living room) and therefore cannot be used outside the home.

Mobile devices, such as smartphones and tablets, provide similar interaction modalities comparing with smart displays and are also able to run virtual or intelligent personal assistants. Moreover, they tend to be much smaller than dedicated smart displays and are usually carried by people everywhere they go (especially smartphones). For these reasons, mobile devices have the advantage of enabling interaction not only inside the home but also remotely.

#### 2.2.1. Smart Home Assistants

As already mentioned above, nowadays, the interaction between smart homes and their inhabitants commonly relies on virtual or intelligent assistants, included in dedicated home devices (smart speakers or displays) or mobile devices used in our everyday life (e.g., smartphones, tablets). These assistants generally enable natural interaction through text or spoken (natural) language.

Some of the most well-known (home) assistants are Amazon’s Alexa [7], Google Assistant [6], and Apple’s Siri [8]. All three of these assistants have similar features, allowing to control various types of devices and appliances (e.g., lighting, security devices, TVs, thermostats, and entertainment systems). Moreover, they have some initiative, including warning when certain sounds and/or motions are detected (complete features usually only available through subscription, or currently limited to some countries), as well as reminding that the lights are turned on or proactively turn them off when no one is home [29,30,31].

These assistants are able to learn and recognize the voices of different users for personalization. However, this functionality is limited to speech interactions. In addition, personalization is generally restricted to the content of calendars, photos, music, etc. rather than the way the information is presented to the user [8,32,33].

Recent research on home assistants includes the proposal of different types of assistants, including a cloud-connect assistant for voice-based controlling and scheduling, integrating speaker identification [34]; a voice assistant running on a Raspberry Pi, which was integrated with a home surveillance system including a camera and face recognition capabilities [35]; and a conversational assistant for supporting smart decision-making, predictive and preventive analytics, based on Telegram, Google Dialogflow, and a Raspberry Pi [36].

Assistants targeting elderly/disabled people have also been proposed, including a home assistant for people with dementia relying on photo notifications [37]; the use of Google Assistant for remote control of home devices by disabled people [38]; an assistant that assists elderly and disabled people in remotely monitoring, accessing and managing home appliances, systems, and surveillance [39]; and an assistant for the blind supporting the Bengali language [40].

#### 2.2.2. Multimodal Adaptive Interaction

Besides a natural and integrated interaction, another important aspect to take into account is the diversity of users of smart homes [17], as well as the dynamic context of interaction [1,19]. If interaction does not adapt to the user, many users can be excluded due to not being able to access their own home. Not accounting for changes in the context may also lead to difficulties or even the inability of having access to the home.

Voice assistants are accessible to people with vision and/or motor difficulties, but cannot be used by the speech-impaired, for example. A possible solution for smart homes that are more accessible to all is multimodal interaction [9,41,42], i.e., interaction through different redundant input/output modalities, which allows the users to choose the most adequate for them and the current context.

An architecture and framework for multimodal interaction was proposed and demonstrated through proof-of-concept applications in smart environments, including smart homes [19,41]. In another contribution, a multimodal interaction system based on three different modalities (eye blinking, speech, and touch) was implemented with the aim of allowing people with limited physical mobility to control the devices and appliances of a smart home [43]. The use of multimodal interfaces was also proposed with the aim of supporting the independent living of the elderly in smart homes [44].

Although some commercial assistants already offer multiple modalities, they tend to present information in the same way regardless of the user or context. This issue can be minimised with adaptive interaction, where the assistant and modalities are able to adapt to the current user(s) and context. This adaptation can be achieved by relying on information obtained from sensors embedded in the device used for interaction and/or dedicated sensors deployed at home [19].

Regarding smart homes, adaptation to the user/context has been used for improving interaction with the home [45,46,47,48,49]. Some contributions relied mainly on a single modality (voice, gesture) and context only [45,48]. In a recent contribution, user awareness based on face identification was proposed for adaptive smart home control through augmented reality [47]. Both user and context awareness (based on user modeling, user-defined home rules, and context data obtained from smart city sensors) was used to personalize the content presented to the user and automate home control [49]. Moreover, a design approach and system were proposed with the aim of adapting the graphic features of the interface and the presented content [46].

## 3. Smart Home Demonstrator

This section presents a demonstrator that integrates our home assistant with various devices (most of them developed for the Smart Green Homes project). We begin by describing the context and defined scenario, and then present the demonstrator’s architecture, as well as some details regarding its implementation.

### 3.1. Context

When developing any kind of system, the developers must always take into account its purposes and the environment that it will be inserted in. The demonstrator presented in this section was designed and developed in alignment with the Smart Green Homes project, and specifically aimed at a pseudo studio apartment deployed at a space built for that purpose.

As the demonstrator’s development had a studio apartment in mind, the divisions of the home are limited to a kitchen, living room, office, and technical room. Each of these divisions is equipped with a few devices that can provide information about their status and/or can be controlled by the user. Table 1 associates each home division to all the devices it contains.

### 3.2. Scenario

The scenario considered for the demonstrator corresponds to a person working remotely from home. The different scenes of the scenario, where the person uses the assistant to interact with the smart home, are described below.

**Scene** **1:** **Select user preferences:** While seating in the leaving room, the user chooses the preferences regarding information presentation, after being identified and greeted by the assistant.
**Scene** **2:** **Consult and control office’s lighting:** Before moving to the office to work, the user relies on text input to get information about the state of the lighting in that division and turns it on, if needed. After finding out about the level of intensity of the light, the user decreases it to a more adequate level for the time of day.
**Scene** **3:** **Consult ambient temperature:** The user checks if the current indoor air temperature is at a comfortable level.
**Scene** **4:** **Consult and control water temperature:** The user also consults the water temperature and asks the assistant to increase it to the user’s preferred value.
**Scene** **5:** **Consult and control kitchen’s instant water heater:** Later, in the kitchen, the user relies on speech to verify if the instant heater of the water tap is turned on. It then turns it on, if necessary, before hand washing.
**Scene** **6:** **Consult water hardness and consult/control water softener:** Back at the office, the user consults the water hardness and then verifies the state of the water softener, using text input. Meanwhile, while working, the user receives an e-mail with an alert regarding an increase in the water hardness, which is now hard. After reading the e-mail on the smartphone, the user once again relies on the tablet to ask the assistant to turn on the water softener.
**Scene** **7:** **Consult air quality and consult/control air purifier:** Through voice interaction with the assistant, the user consults the homes’ air quality at that moment. If the quality is considered unhealthy, the user turns on the air purifier, after confirming that it is currently turned off.

### 3.3. Architecture

**Figure 1:**
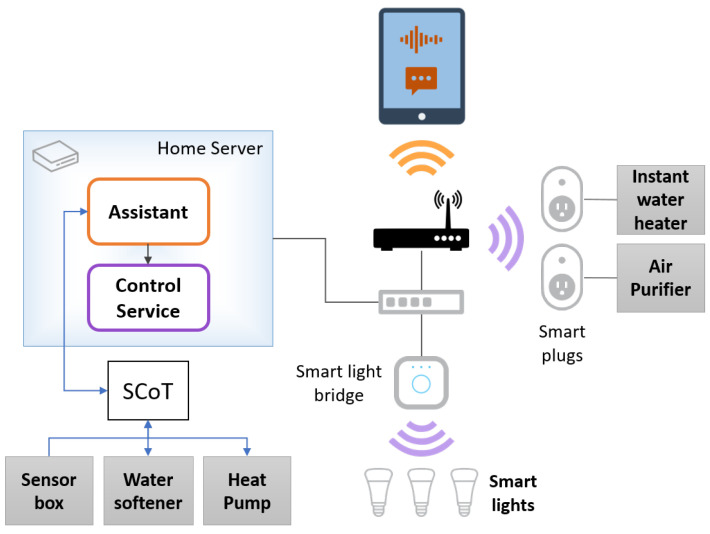
Architecture of the demonstrator, including the home assistant (running in the home server and accessible through a mobile device, such as a tablet) and the different home’s devices.

The demonstrator’s architecture, shown in Figure 1, includes the home assistant (running in the home server) and the appliances and devices installed in the different home divisions (indicated in Table 1).

Users interact with the home using the assistant on a mobile device, such as a tablet (web browser). Some of the appliances (instant water heater and air purifier) are plugged into a smart plug (TP-Link), while the smart lights (Philips Hue) are connected to a bridge through WiFi. The plugs and bridge are connected to the home’s local area network (LAN) through WiFi and Ethernet, respectively. The assistant communicates with them through a control service, also running in the home server. Communication with the remaining appliances/devices relies on SCoT (Smart Cloud of Things) [50].

### 3.4. Implementation

The assistant consults and controls the smart lights and plugs using the control service. This service corresponds to a web service we implemented relying on the API (Application Programming Interface) libraries for Node.js that allow communication with the Philips Hue Bridge (node-hue-api) and the TP-Link plugs (tplink-smarthome-api).

For each of the other devices, their information is kept up to date by listening to one or more RabbitMQ queues, using the Advanced Message Queuing Protocol (AMQP). Information is published in the queue(s) by SCoT [50], which receives it periodically from the corresponding device. In the case of device control, the assistant sends a control request to SCoT, which relays it to the appropriate device.

Besides the conversational capabilities, the assistant also incorporates some alerting capabilities based on the information received from devices such as the sensor box (indoor air quality) and the water softener (water hardness). For example, when CO${}_{2}$, CO and/or, NO${}_{2}$ exceed a given threshold, an alert is sent to the e-mail of the home user(s) informing that the indoor air quality is poor and suggesting that the air purifier should be turned on. An alert is also sent if the water’s hardness decreases below a given threshold, with the user(s) receiving an e-mail warning them of this and advising them to turn on the water softener. To facilitate the demonstration of this capability, changes in the air quality and water hardness can be simulated through a simple web application we implemented for that purpose.

## 4. Home Assistant

In this section, the home assistant is described in more detail, including the defined requirements, used method and main iterations of its development, overall architecture, home information structuring, user and context models, and adaptation of presented information to the user and context.

### 4.1. Requirements

The main requirements defined for the home assistant are the following:

- Being accessible to all inhabitants (from young to old, with or without disabilities), anytime and anywhere;
- Enabling a unified view of the home, by having capabilities that allow the storage and access to rich relevant information on the home and its devices (backend support to complex queries on information, profiting from semantic knowledge bases and semantic search);
- Providing redundant use of interaction modalities;
- Supporting European Portuguese;
- Having enhanced multi-turn conversation capabilities (e.g., reminding information from previous turns);
- Supporting mixed user and system initiative, to make the system more proactive (e.g., take initiative in situations involving alerts);
- Having enhanced user and context awareness and adaptation.

### 4.2. Development Method and Main Iterations

The home assistant was implemented by adopting an iterative design and a development for all approach. Initially, a family of Personas was defined together with different accessible scenarios to understand the needs of the different potential users of a smart home (e.g., younger and older people, people with one or more disabilities) [27]. Based on this information, an initial proof-of-concept assistant was developed and demonstrated using a simulated home.

Next, a study with twenty participants was carried out [12], providing more important capabilities to consider: remote control, home state report, spoken communication, querying and control of appliances and lights, activity scheduling for appliances, control of the temperature for appliances (e.g., water heater) and home divisions, and obtaining consumption information. A new version of the assistant was implemented considering these results, which was then evaluated by six participants, who carried out several tasks using the assistant together with a simulated home [28]. The obtained results allowed the identification of some aspects that could be improved, such as the onboarding logic and dialogue manager, as well as the addition of initiative to start a conversation and notifications in critical situations, which were addressed in a new iteration.

Recently, the assistant was evolved by further adding the capability of adapting the interaction to the user and context [51]. For integration and evaluation in a real scenario, two of the objectives for this paper, the assistant’s conversational capabilities were improved, with special attention being paid to the capability of multi-turn conversation, which relies on the context from previous turns to disambiguate, allowing simpler user inputs, or ask for more information in the following turn when only partial information is provided. The assistant was then integrated with real devices, resulting in the demonstrator described in Section 3. This demonstrator was used to evaluate the assistant by performing tests with end users.

### 4.3. Assistant Architecture

**Figure 2:**
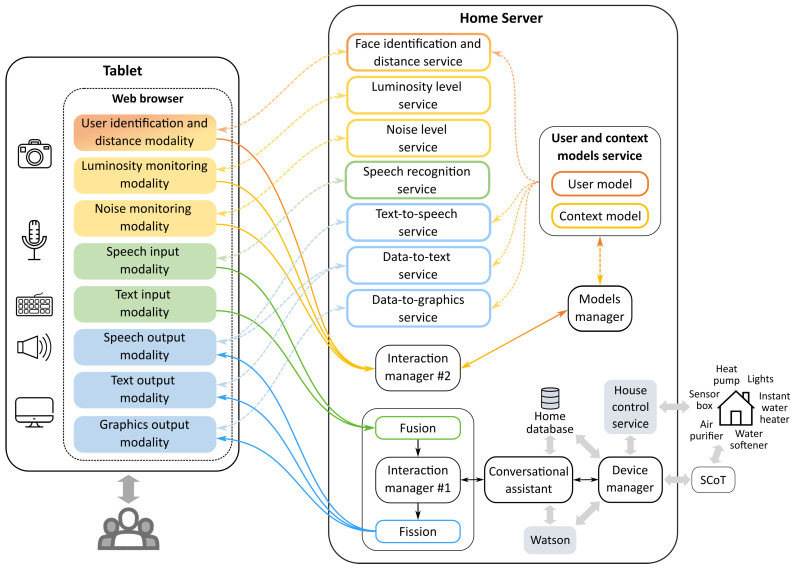
Architecture of the home assistant, including all the components that enable multimodal adaptive interaction with a smart home.

The architecture of the assistant, illustrated in Figure 2, is an extension of the state-of-the-art interaction architecture AM4I (Adaptive Multi-platform Multidevice Multilingual Multimodal Interaction) [19], which is aligned with the multimodal interaction architecture proposed by the W3C (World Wide Web Consortium) [52]. One of the main advantages of this architecture is that it is modular and distributed, with decoupled components, enabling future modifications and/or extensions.

The assistant’s architecture includes different types of modalities, including redundant input and output modalities, as well as passive/implicit modalities. Input is managed by a fusion engine, which fuses the user inputs if needed and sends the result to an interaction manager (interaction manager #1—IM#1). The latter sends the fused input to a conversational assistant, which extracts the relevant information using the natural language understanding (NLU) capabilities of IBM’s Watson Assistant [53].

Based on the extracted information, the conversational assistant generates a response, after querying the home database and/or controlling the home’s devices, through the device manager (running one thread per device), which uses the house control service or SCoT. The generated response is sent to IM#1, which then forwards it to a fission engine. This engine decides which output modality(ies) should be used to present the information to the user and then sends the information only to those modalities. For now, the output modality choice is based only on the used input modality.

Each output modality is responsible for adapting the received information to the user and context, relying on one or more services (text-to-speech, data-to-text, and/or data-to-graphics services). These services rely on another service, the user and context models service, to obtain relevant information on the current user and context that allows choosing the most appropriate output properties (more details in Section 4.6).

The smart home’s users and context information is stored in two different databases: as a user model and a context model, respectively. These models are updated by the passive/implicit modalities using a models manager application. The communication between them is managed by a second IM (interaction manager #2). The latter is also used to intermediate communication between all different types of modalities (not indicated in Figure 2 for simplicity).

### 4.4. Smart Home’s Information Structuring (Home Database)

To enable an integrated view of the home, we adopted Design for Data and a semantic-based approach for all the information [1,12]. This approach allows the storage of all relevant information on the home and its devices in an organized way, as well as enables richer queries from the users.

More specifically, all data provided by the home devices are stored in the home database, relying on the domain ontology proposed by our group [1,12], and information is retrieved from the database using SPARQL queries. The ontology includes the following classes: Partition, for describing home divisions; Device, for home appliances and devices present in each home division; Resource, representing resources consumed by each device, such as water, electricity, or gas; Consumption, representing the consumption of a given resource by a device; Error, for errors associated with a device; and Issue, to describe the occurrence of a specific error for a given device.

### 4.5. User and Context Models

The user and context models are essential for user and context awareness, which enables interaction adaptation. Each model was implemented as a document database, namely, MongoDB [54], to allow for future extensions. Both models can be accessed or updated through the user and context models service (Figure 2).

The user model includes personal, health, security, and preferences data of a given user. The personal data include the user’s name and birth date. The health data include the vision and hearing capabilities. The security data include a face encoding, which is used for user identification. The preferences include the preferred output modality and different properties related to text (font size, font colors, and background colors) and speech (rate and volume) outputs. It also includes context information at the moment the preferences were saved (user distance, noise level, and luminosity level). Please refer to the work in [51] for more details.

The context model represents the relevant context data. As multiple users can interact simultaneously with the system using different devices, this model includes the current context information for each device. The context includes the identity of the device’s current user, user distance (relative distance between the current user and the device), and associated environment conditions (noise and luminosity levels).

### 4.6. Output Adaptation

Besides providing redundant input and output modalities, accessibility is further enhanced in the assistant by having output modalities that can adapt themselves according to the user and context. The implemented solution builds on previous work by our group on output adaptation [55,56].

Regarding the text output modality, it selects the most appropriate text properties (font size and colour, and background colour) for presenting the text on the device’s screen. In our solution, the graphics output modality presents graphs of time series data, also in the device’s screen, with the following properties adapted: background, time series, and font colours, as well as the font size. The speech output modality uses the device’s speakers to play the output as audio, with adapted speech rate and volume.

The output properties are chosen based on adaptation rules, described in [51], according to the current user’s preferences or characteristics (age, vision, or hearing capability), and the user distance and/or the environment’s luminosity or noise level.

As already explained above, all relevant user and context information is stored in the user and context models. Most user data are provided by the users themselves. The context data and the identity of the current user(s) are updated by the passive/implicit modalities. The user identification and distance modality uses the device’s camera to identify the current user and obtain the user distance. The level of the environment’s luminosity and noise is measured by the corresponding monitoring modalities using the device’s camera and microphone, respectively. Each of these modalities relies on an associated web service to obtain the described information based on the sensor’s input. More details on output adaptation can be found in [51].

## 5. Evaluation

Using the implemented demonstrator, the home assistant was evaluated by carrying out tests with end users. Aligned with recent similar evaluations [57], the performed evaluation involved the visualisation of a video, which shows the assistant being used by a person in the context and for the scenario described in Section 3. The focus of this evaluation was the integrated multimodal interaction with real smart home devices (using text and speech input/output). The adaptation of interaction was limited to the identification and greeting of the person by the assistant and to the setting of text output preferences by the person.

The whole evaluation was carried out using a web application implemented for this purpose. After reading an overview of the evaluation and the informed consent (and agreeing to participate voluntarily in the experiment), the participants answered the pre-questionnaire. Next, they watched a video presenting the assistant, followed by another video showing the assistant’s demonstration. Finally, the participant answered the two post-questionnaires.

### 5.1. Participants

The evaluation described above allowed for remote participation. Volunteers were recruited among the communities of Bosch Termotecnologia and University of Aveiro, as well as personal contacts of people involved in the Smart Green Homes project. The participants could also share the evaluation information with their contacts. The evaluation was available for a week and a half in March 2021.

The inclusion criteria were being 10 years old or more, having access to a computer with Internet and web browser, and understanding European Portuguese. No exclusion criteria were considered concerning the knowledge and usage of mobile devices and virtual/home assistants.

The number of participants was 70 (45 male, 25 female) subjects, with an age mean ± standard deviation [minimum, maximum] of 36.7 ± 12.0 [10.0, 73.0] years old. All participants usually use a mobile device (e.g., smartphone, tablet). Most of them (59 participants—84%) use it very frequently (i.e., more than 10 times a day). The remaining 4 and 7 participants use it either 2 to 5 times per day or 5 to 10 times per day, respectively.

About the use of virtual/home assistants, 70% of the participants do not usually use a virtual assistant. Of those who use it (30%), most (67%) use it at least once a day, with the same number of participants (7) using it 1 time or more than 1 time a day. The remaining participants use it only once a week (5 participants—24%), or 0 to 1 time per month (2 participants—9%).

Sixty-four percent of the participants have never used a smart speaker with an integrated assistant (e.g., Google Home and Amazon Echo). A larger percentage (80%) do not usually use an assistant to interact with their home. Of those using a home assistant (20%), all except one use it at least once a day, with 9 and 4 participants (64% and 29%) using it more than 1 time per day and 1 time per day, respectively. The other participant uses the home assistant 0 or 1 time per month.

### 5.2. Evaluation Instruments

Besides the personal computer of the participants, the instruments used in the evaluation include all the content of the web application used to carry out the evaluation. All material was written in European Portuguese, as it is the language of the assistant.

The evaluation’s overview includes the main steps involved in the evaluation and their estimated duration. The informed consent describes the evaluation’s main objective; provides a brief description of the evaluation and its estimated duration (25 min in total); gives information regarding data dissemination, confidentiality, and volunteer nature of participation; and includes the contact of those responsible for the evaluation.

The pre-questionnaire consists of questions regarding the participant’s gender and age, as well as the daily use of mobile devices and virtual (home) assistants. The complete question list is included in Appendix A. The responses to these questions were used to obtain the characterization of the participants presented in the previous subsection.

The presentation about the assistant is a narrated video, which aims at familiarizing the participant with home assistants and more specifically with our implemented home assistant and its capabilities, as well as the used demonstrator.

**Figure 3:**
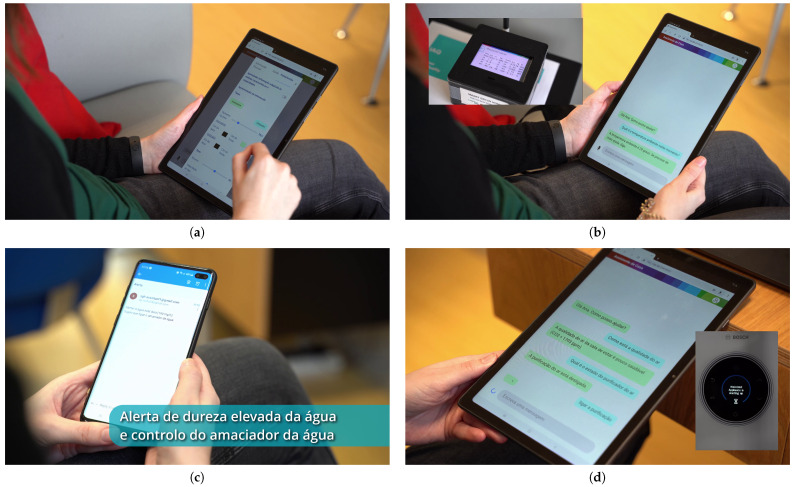
Screenshots of the video with the assistant’s demonstration (in European Portuguese), corresponding to the following tasks: (**a**) choosing output preferences; (**b**) consulting the ambient temperature (measured by the sensor box); (**c**) receiving an alert regarding water high hardness, which is later followed by turning on the water softener; and (**d**) turning on the air purifier, after being informed that the air quality is unhealthy.

The video with the assistant’s demonstration shows a person using our assistant, in a tablet, to interact with the smart home (in this case, the demonstrator) using natural language, relying on either text or speech input/output, according to the scenario described in Section 3.2. Some screenshots of this video are shown in Figure 3.

The first post-questionnaire has diverse questions on the assistant demonstrated in the video, including questions typically used to evaluate a product, which were adapted to the assistant. We also included questions that are more specific to our assistant, such as questions concerning home interaction and the assistant’s capabilities. The complete question list is included in Appendix B.

The second post-questionnaire corresponds to the 118-word test (Microsoft Product Reaction Cards) [58]. This test was developed as part of a “desirability toolkit” created to evaluate the aspect of desirability resulting from a user’s experience with a product, by choosing descriptive words or multi-word expressions from 118 reaction cards [59].

The complete word list (in English) is presented in Table 2, where each word is associated with a positive, neutral, or negative sentiment, indicated using red, black or green font color, respectively. The sentiment was decided based on the results obtained with the R package LexiconPT [60] for each word (in Portuguese). In the case of a multi-word expression, or when the word was not found, words with similar meaning were considered. For words with inconclusive results, we additionally took into account their meaning in the context of our assistant. Regarding neutral words, the decision was mainly based on them having both a positive and negative meaning depending on the point of view. The Portuguese translation of the words/expressions used in the evaluation was obtained from the work in [61].

The participant is first asked to choose five or more words from the set of 118 words presented in Table 2, which are shown as “cards” that can be (de)selected, in groups with a maximum of 10 words. The words are presented in a random order for each participant. The words chosen in the initial selection are then presented in a final step, where the participant is asked to choose only five of those words.

## 6. Results

This section presents the evaluation results, including those related to the simplification of interaction with the home, usefulness of the assistant’s functionalities, accessibility, interest in using/buying the assistant, its desirability, and overall impression.

### 6.1. The Assistant Enhances Interaction with the Home’s Sensorized Ecosystem

**Figure 4:**
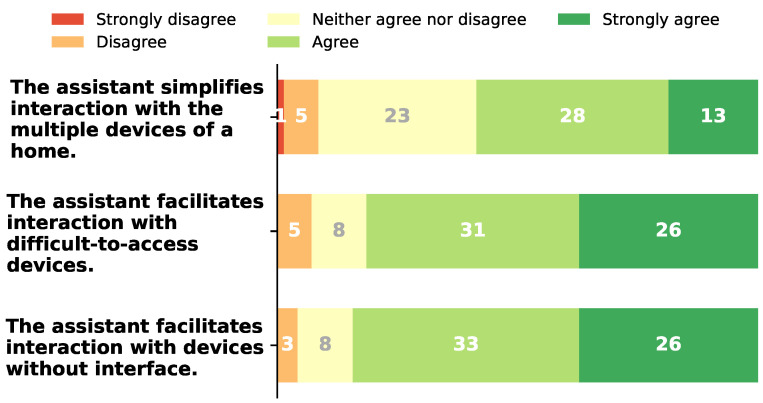
Results for the questions concerning the interaction with the home’s devices using our assistant.

Figure 4 shows the results for the questions corresponding to whether the assistant simplifies the interaction with the multiple home’s devices, and if it facilitates the interaction with devices that are more difficult to access (e.g., water softener and heat pump) or that do not have an interface (e.g., instant water heater).

More than half of the participants (59%) agree or strongly agree that the assistant simplifies the interaction with multiple devices. Most of the remaining participants gave a neutral answer (33% of all participants), with only 7% and 1% disagreeing and strongly disagreeing, respectively.

The results are even more positive when considering the interaction with difficult-to-access devices or devices without an interface, where 81% and 84% of the participants, respectively, agree or strongly agree that it is easier with our assistant. No one strongly disagreed, with only 7% and 4% disagreeing and 11% giving a neutral answer.

### 6.2. Automatic Alerts Considered as the Most Useful Functionality

**Figure 5:**
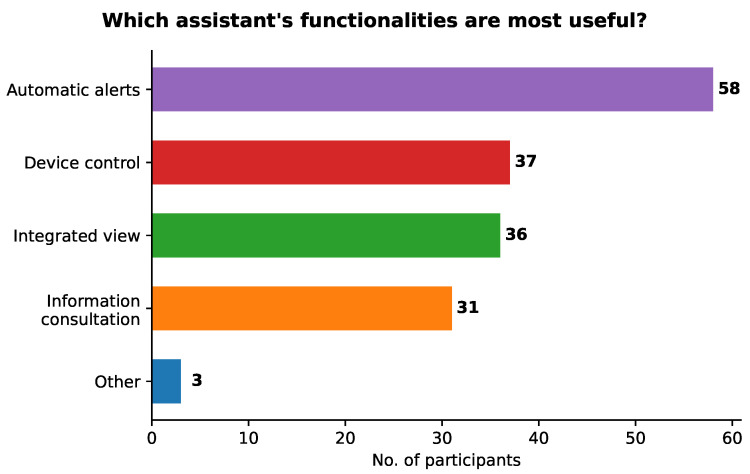
Results for the question on the most useful assistant’s functionalities.

Figure 5 presents the results for the question about the most useful functionalities of the assistant. The capability of automatic alerts was the most frequently chosen by 58 out of the 70 participants (83%). Approximately half of the participants indicated the control of devices and an integrated view of the home in a single application as the most useful functionalities (53% and 52%, respectively). Although allowing the consultation of information on the home was the less selected functionality, it was still considered as the most useful by 31 participants (44%).

Note that each participant could choose one or more functionalities, as well as the option “Other”. Three participants chose that option. One of them indicated the fact that the assistant can be used by different users, with different profiles, in various contexts, which is extremely useful for people with motor difficulties. The second participant mentioned the possibility of interaction through diverse modalities. The other participant answered “I cannot find something that is unique and different from what already exists.” Although this is a valid opinion, it does not answer the question, which asked about the usefulness and not the uniqueness of the assistant’s functionalities.

### 6.3. The Assistant Is Generally Considered to Be Accessible

**Figure 6:**
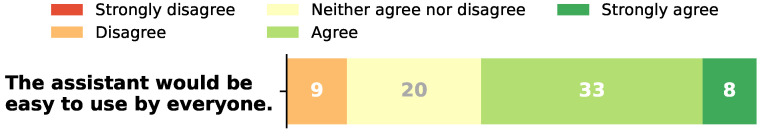
Results for the question on whether the assistant would be easy to use by everyone.

The results for the question on the assistant’s ease of use by everyone are presented in Figure 6. More than half of the participants (59%) agree or strongly agree that the assistant would be easy to use by everyone. Twenty-nine percent answered neutrally, and only 13% disagreed. No participant strongly disagreed. These results are very positive, as the main focus of the evaluation was not the adaptation of interaction to the user.

### 6.4. Participants Manifested Interest in Using and Buying the Assistant

**Figure 7:**
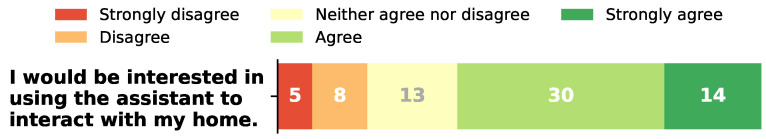
Results for the question about the interest of the participants in using our assistant to interact with their homes.

Regarding the interest in using our assistant, as can be seen in Figure 7, more than half of the participants (63%) said they would be interested or highly interested in using the assistant to interact with their homes. Nineteen percent showed neither interest nor disinterest, indicating that an important percentage of users may be persuaded after making some improvements to the assistant. The remaining 11% and 7% participants showed little or no interest.

Of those who answered positively (agree or strongly agree) or neutrally (neither agree nor disagree) to the question concerning the interest in using the assistant, 61% believe they would use the assistant at least once a day, with most (42%) using it multiple times per day. The remaining 17 and five participants (30% and 9%) said they would use it a few times a week or month (no one thinks they would use it only a few times per year).

**Figure 8:**
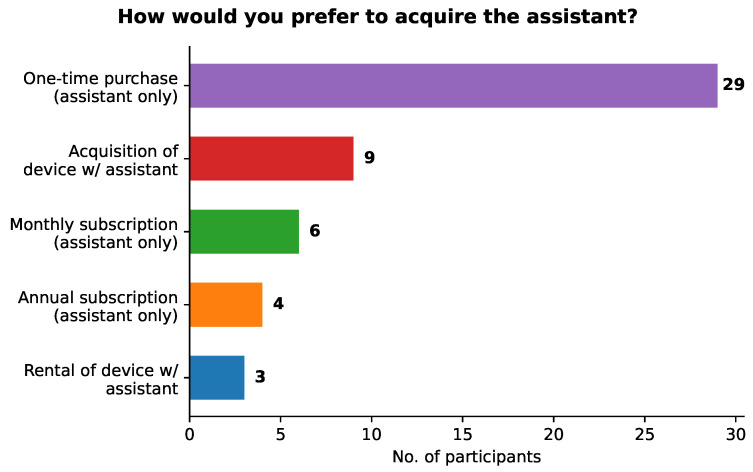
Results for the question concerning the preference for the acquisition of our assistant.

As for purchasing the assistant, most of those participants (72%) answered that they would pay (15 participants—21%) or might pay (36 participants—51%) for it. Considering only those who would pay or might pay for our assistant, their answers to the question about how they would prefer to acquire the assistant are shown in Figure 8. It is clear from this figure that the preference for the majority of the participants is to buy the assistant only through a one-time purchase (57%). The second most chosen option was acquiring a device, such as a tablet, with the assistant already installed (17%), followed by a monthly subscription of the assistant only (12%). The annual subscription of the assistant only and the rental of a device with the assistant installed were chosen by the remaining participants (8% and 6%, respectively).

As for the amount they would be willing to pay, considering all acquisition preference options except monthly subscription, 35 out of 45 participants (78%) chose an amount between 10 and 1000 €, with most of them (58%) opting for 10 to 100 €. The remaining 10 participants chose the option of less than 10 €. No one opted for more than 1000 €, which was already expected. It is a positive surprise that most participants chose the option of paying 10 to 100 €, especially when given the possibility of a lower amount.

For the six participants who chose the possibility of buying the assistant through monthly subscription, four of them (67%) said they would pay 5 to 10 €, with the remaining two choosing less than 5 €. These values are in line with the prices of monthly subscriptions for the most well-known online services.

### 6.5. The Assistant Is Overall Highly Desirable

The assistant’s desirability was evaluated using the 118-word test, which involves an initial and final selection of words/multi-word expressions. The mean ± standard deviation [minimum, maximum] number of words initially selected per participant was of 19.9 ± 11.9 [5, 60]. One-hundred-and-six out of the 118 different words were selected by at least one participant. Considering the words’ sentiment, positive words were chosen more often (mean of 17 times), followed by neutral (10 times). Negative words were selected much less frequently (only 4 times on average).

In the final selection, each participant has to select exactly five words from their initial selection. Overall, 86 different words were chosen at least once. The number of times each word was selected decreased, with a mean of 5, 3, and 2 times for positive, neutral, and negative words, respectively. The difference between neutral and negative is smaller comparing with the initial selection, which can be explained by the fact that the number of words that can be selected in the final step is much more limited.

**Figure 9:**
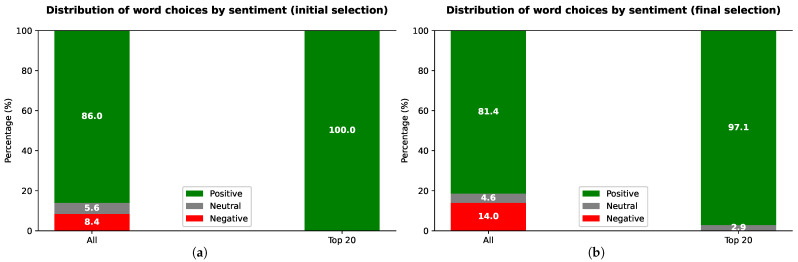
Word distribution according to their sentiment (positive, neutral, or negative), considering all words and only the twenty more frequently chosen words (top 20), for the (**a**) initial and (**b**) final selection of the 118-word test.

Figure 9a,b presents the distribution of word choices according to their sentiment, for the initial and final selections, respectively, when considering all word choices and only the twenty most often selected words (top 20).

In the initial selection, positive words were chosen more often, with 86% of all choices corresponding to positive words and only 8% to negative words, when taking into account all words (Figure 9a). To correctly interpret these results, note that in the set of 118 words, 60%, 7%, and 33% are positive, neutral, and negative, respectively. Even considering this proportion, the achieved results are quite positive. Results are even better if only the top 20 words are considered: all choices are positive.

The percentage of negative words is higher in the final selection than in the initial one (14% vs. 8%). However, the percentage of positive words remains much higher than negative words (81% vs. 14%—Figure 9b). Moreover, the top 20 words are mostly positive (97%) and again do not include any negative words.

More detailed results concerning the selected words for both initial and final steps are presented below.

#### 6.5.1. Initial Word Selection

**Figure 10:**
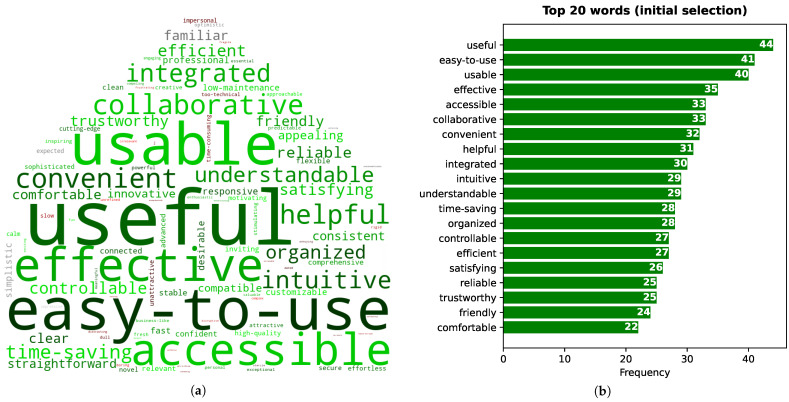
Word cloud considering all chosen words (**a**), and word frequency (i.e., number of times a word was chosen) considering only the twenty more frequently chosen words (top 20) (**b**), for the initial word selection of the 118-word test. In the word cloud, a larger font size means that the word was selected by more participants (higher frequency).

The frequency (i.e., the number of times a word was selected) for all words selected in the initial selection is represented in Figure 10a by a word cloud, where a larger font size corresponds to a higher frequency. The “words” corresponding to multi-word expressions were hyphenated (i.e., spaces replaced by a hyphen) to simplify the creation of the word cloud. Figure 10b shows the frequency for each top 20 word.

From Figure 10b, we can see that the three most frequent words, which were selected by more than half of the participants, are the following: “useful”, “easy-to-use”, and “usable”. These results are in accordance with the results presented above, where the various capabilities of our assistant were found to be quite useful by most participants, and most participants agreed that the assistant would be easy to use by everyone.

The word “accessible” is part of the top 6 words, having been chosen by 47% of the participants. This result is very relevant, as accessibility is one of the main concerns of the implemented assistant. Another word that stands out is “integrated” (chosen by 43% of the participants), as one of the main aims of the assistant is to provide an integrated view of the different devices of a home. This choice is also in accordance with the results presented above, where the capability of an integrated view was considered as the most useful one by around half of the participants, and as useful as controlling the home’s devices.

Other relevant top 20 words include “intuitive”, “understandable”, “time-saving”, “reliable”, “trustworthy”, and “friendly”. When considering all word selections, some of the negative words that were chosen more often were “unattractive” (46th most chosen), “impersonal”, “slow”, “time-consuming”, and “too technical” (61st) (words in shades of red in Figure 10a).

#### 6.5.2. Final Word Selection

**Figure 11:**
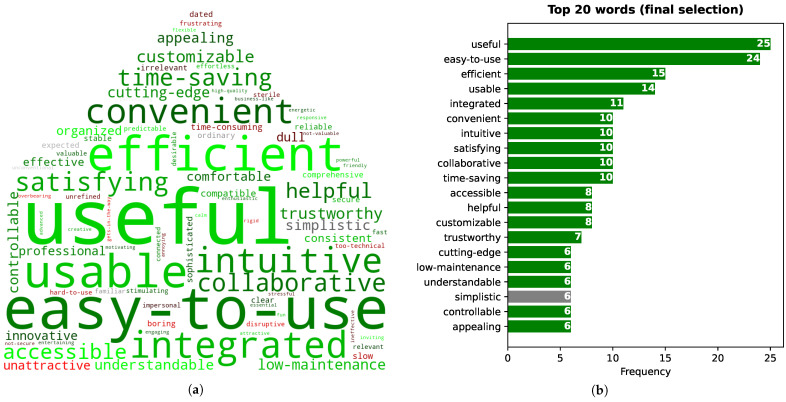
Word cloud considering all chosen words (**a**) and word frequency (i.e., number of times a word was chosen) considering only the twenty more frequently chosen words (top 20) (**b**) for the final word selection of the 118-word test. In the word cloud, a larger font size means that the word was selected by more participants (higher frequency).

Results similar to those presented above for the initial word selection are shown in Figure 11a (word cloud) and Figure 11b (frequency of top 20 words). The two most frequent words are the same for the initial and final steps (“useful” and “easy to use”). However, the difference between them and the third most frequent word (“efficient”) is considerably higher in the final selection. Nevertheless, all words from the initial top 20 are also in the final top 20 (including “integrated”, as the fifth most frequent word), except for five words (“effective”, “organized”, “reliable”, “friendly”, and “comfortable” from the initial selection). One of the new words is “customizable”, indicating adaptation to the user, which also contributes to accessibility. Another new word is “cutting-edge”, which indicates that some participants believe our assistant is innovative. The remaining new words are the following: “low-maintenance”, “simplistic” (the only neutral word in the final top 20), and “appealing”.

As can be seen from Figure 11a, the most frequently chosen negative words (in shades of red) are “dull” and “unattractive” (23th and 25th most chosen), followed by “time-consuming” and “slow” (30th and 32th). This set of words is similar to those observed in the initial word selection. In this final selection (where each participant could only select 5 words), these words were chosen by only 6–7% of the participants.

### 6.6. Global Impression of the Assistant Is Positive

**Figure 12:**
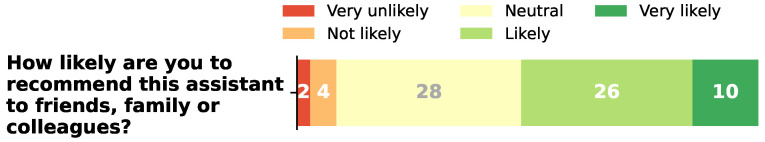
Results for the question about how likely the participant is to recommend our assistant to friends, family, or colleagues.

**Figure 13:**
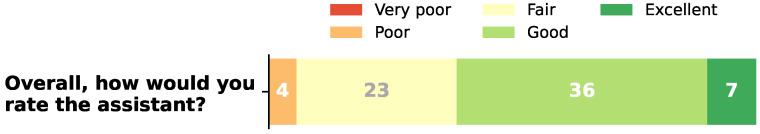
Results for the question on how the participant would rate the assistant overall.

The global impression of the assistant was positive, as can be seen in Figure 12 and Figure 13, which show the results concerning the likelihood of participants recommending the assistant to friends, family, or colleagues, and the assistant’s overall rating, respectively.

Approximately half of the participants (51%) would likely or very likely recommend it to friends, family, or colleagues. Of those who would not (very) likely recommend it, 82% (28 out of 34) gave a neutral answer.

The overall rating of the assistant obtained even better results, with 61% rating it as good or excellent, and only 6% rating it as poor. The remaining participants gave a neutral rating, with none considering it to be very poor.

When it comes to comparing our home assistant with other similar solutions, 33% and 19% of the participants said that our assistant is a minor improvement or similar to existing solutions, respectively. Five percent think that the existing solutions are more adequate. However, most (43%) considered they did not have enough knowledge about alternative solutions to make that comparison.

## 7. Discussion

The obtained evaluation results show a positive global impression towards our assistant, with 61% of the participants rating it as overall very positive or positive, only 6% as poor, and no one as very poor.

When asked to compare the evaluated assistant with similar available solutions, more than half of the participants said our assistant is only a minor improvement or similar to existing solutions, and a small percentage believe that existing solutions are more adequate. These results may be due to a limited demonstration of some of the assistant’s capabilities, including the adaptation to the user and context, and especially the assistant’s initiative involving alerts (only one type of alert was shown in the demonstration video during the evaluation), which was chosen most often as its most useful functionality.

Moreover, it is important to consider that approximately 4 out of 10 participants thought they did not have enough knowledge about alternative solutions to compare our assistant to them. Additionally, 70% of the participants do not usually use a virtual assistant, and an even larger percentage of 80% do not usually use an assistant to interact with their home. Therefore, some of the participants who did compare our assistant to existing solutions probably have little experience with virtual/home assistants, which may have negatively influenced the results.

Regarding the main objective of exploring if an assistant like the one implemented by us could enhance interaction with the various devices of a smart home, the obtained results show that our home assistant has the potential to be used to simplify this interaction, especially with devices that are difficult to access or do not have an interface.

All functionalities of the assistant were considered as the most useful by at least 44% of the participants. Information consultation was the least chosen, maybe because obtaining information from an assistant is one of the most common functionalities. Device control and an integrated home view were both chosen by approximately half of the participants as the most useful functionalities, showing that having an integrated view of all home’s devices is as important as being able to control them. Automatic alerts stood out as the most useful for 83% of the participants, suggesting that greater attention should be given to it in future iterations of our assistant. Two of the participants also highlighted the fact that our assistant provides different forms of interaction, and that it can be used by diverse users in various contexts.

These opinions are reinforced by the results related to the assistant’s accessibility, where approximately 60% of the participants agreed or strongly agreed that the assistant is easy to use by everyone (no one strongly disagreed). These results are interesting, as the main focus of the present study was not the adaptation of interaction to the user. However, the possibility of adjusting text output’s preferences and the use of both text and speech input/output, which were demonstrated in the evaluation’s video, may have contributed to the positive responses. An improvement and greater focus on interaction adaptation to the user and context in future evaluations can lead to even better results concerning the assistant’s accessibility.

The obtained results also showed a relatively high interest of participants in using our assistant to interact with their smart homes (63% would be interested or highly interested), with most of them saying they would use it frequently (at least once a day). Moreover, a big percentage (72%) of the participants that were neutral or (very) positive about using the assistant said they would pay or maybe pay for it. The most popular way of acquiring the assistant is a one-time purchase of the assistant only, with most participants being willing to pay between 10 and 100 €, which is surprising as they could choose a lower amount (<10 €). These results indicate that our assistant has value to the end users.

The results of the 118-word test show that our assistant is overall highly desirable, confirming the generally positive attitude towards the assistant considering the remaining evaluation results. A large percentage of all words choices are associated with a positive meaning (86% or 81% for the initial or final selections, respectively). When considering only the 20 more frequently selected words, the percentage of positive word choices is even higher (100% or 97%). Furthermore, the 20 most frequently chosen final words include words related to its usefulness and ease of use (e.g., useful, easy-to-use, usable, time-saving, helpful, and understandable). In accordance with the other obtained results, the final top 20 words also included words reflecting the integrated view of a smart home in a single application (integrated), as well as the accessibility and adaptability (accessible, customizable) and innovation (cutting-edge) of our assistant.

The 118-word test results also included some negative words (e.g., unattractive, dull, time-consuming, slow). Although they were much less frequently chosen, comparing with some positive words, they also provide important information on some aspects that can be refined in the future.

The evaluation’s results also allowed to gain some insight on the sensorized part of the smart home and its contribution to the proposed system. For example, the interaction with devices that are difficult-to-access, and especially those without any interface, is greatly facilitated due to the integration of sensors and actuators. Other devices that stand out are those that allow measuring certain characteristics of the indoor air (e.g., CO${}_{2}$) and water (e.g., hardness), as they allow the system to have more initiative (for example, by sending alerts when it detects changes in those characteristics).

Despite the overall positive results of our study, the assistant’s functionalities are still limited, mainly due to the lack of available sensors that provide additional relevant information, such as the consumption of different resources (e.g., water, gas, and electricity) by each appliance/device. This type of information would further enhance the system’s initiative (e.g., informing the users about increases in their consumption) and potentially lead to savings, both financially and environmentally. Other sensors that would allow to increase the assistant’s functionalities include motion detectors, smart door locks, and other kitchen appliances (e.g., refrigerator and washing machine).

Regarding home assistants, while many authors focused on a specific group of people, such as the elderly and/or disabled [37,38,39,40,43,63], our assistant is meant to be accessible to all home inhabitants, from younger to older, with or without temporary/permanent limitations. Furthermore, most works mainly aim at device control and/or home automation [38,43,63,64,65]. In contrast, our system goes beyond simple control or automation, by allowing obtaining rich relevant information on the home and its devices. It additionally has initiative, i.e., alerts the home users when it detects certain situations, such as poor air quality and water hardness above normal. In general, few or no details are given in the relevant literature about the structuring and handling of data regarding the home and all its devices and appliances. Some works just mention the use of a database [34,36,37,44,64]. Only two contributions adopted a semantic approach similar to ours [49,66], by using an ontology, although none of them include an assistant. As for system initiative, the few examples found in the literature are limited to a single type of alert: fire alarm according to information provided by flame and temperature sensors [64]; use of a light sensor to inform the blind users about day/night [40]; alert when an intruder is detected in the context of surveillance [39]; alert when a person with dementia leaves food in the cooker longer than needed [37].

Concerning the conversational capabilities of assistants, many contributions do not provide enough details, with most only mentioning speech recognition and synthesis [35,40,63]. An outlier is the conversational assistant proposed by Salvi and coworkers [36], which relied on Google’s Dialogflow platform. Other authors used commercially available assistants, such as Google Assistant [38,65] and Alexa [64]. In regard to the language(s) covered by the assistants proposed in the literature, the most common is English [34,35,36,37,38,39,64]. Only a few support different languages, such as Spanish [43], Indonesian [63], or Bengali [40]. Even counting our previous work [1,27,28,51], European Portuguese support is quite rare.

The multimodal nature of our system (speech, text, and graphics) is aligned with several works [34,36,37,38,39,40,43,44]. Some authors relied on user and/or context awareness for interaction adaptation [45,46,47,48,49], but this capability was not integrated into a home assistant. In contrast with these works, we used both multimodal and adaptive interaction to improve the accessibility of smart homes. The closest match to our work is the adaption of multimodal interaction by Contreras-Castañeda and coworkers [43], which is limited to adaptation to the user and relies only on parameters defined by the users themselves.

Works where the assistant is integrated and deployed in a real home scenario are not easily found in the literature, with many being demonstrated only in a laboratory environment [39,40,43,63,64]. Exceptions include the assistants proposed in [34,35,36]. However, they did not evaluate the assistant as a whole with end users.

The evaluation of home assistants frequently favors more technical tests related to speech identification and/or interaction response times [34,36,38,43]. An exception is the evaluation performed by Rahman and coworkers [40], which uses the System Usability Scale (SUS), a highly graphical user interface (GUI) oriented evaluation tool [67]. Kocaballi and coworkers compared different questionnaires for evaluating the user experience in conversational interfaces, including SUS and questionnaires specific for voice interfaces, and recommend using multiple questionnaires for a more complete evaluation [68]. Therefore, we adopted a questionnaire tailored to our assistant, including some important aspects (e.g., product-related questions), and complemented it with a test to assess the desirability of our assistant. Although we did not find any contribution using a similar evaluation of a home assistant, our approach provides more comprehensive insights on the system comparing with other approaches.

Considering works minimally related to ours, but not directly comparable, Rahman and coworkers used SUS to evaluate their assistant with 15 blindfolded subjects [40]. They report positive results, with a high percentage of participants endorsing or strongly endorsing the solution, but not being clear how this endorsement was derived from the SUS scores. As a very recent evaluation example, a system targeting the interaction of elderly people with a smart home (not including a conversational assistant) was evaluated with 10 users (over 60 years old) who performed 12 tasks [69]. The authors relied on different evaluation tools, including a Likert scale (1 to 5) for rating each task, criticism and suggestion reporting, SUS, and Self Assessment Manikin (SAM) questionnaire. Seventy-five percent of the tasks were considered very easy and the obtained SUS score was 68.75. Most participants were extremely satisfied with their system and also felt very motivated (the feeling of control had a neutral rating). Moreover, the authors identified some usability problems (difficulties with device control, GUI navigation, etc.). These results are, in general, aligned with ours, as the results were overall positive, but also allowed to identify some aspects that need improvement in the future. Additionally, there are other contributions dedicated to the evaluation of commercially available voice assistants [16,67,70]. However, they do not perform the evaluation in the specific scenario of a smart home, considering mainly general commands related to the weather, shopping, alarms, directions, music, news, etc.

## 8. Conclusions

The main objective of our work is to develop accessible, usable, useful, and easy-to-use systems. To achieve this objective, in this contribution, we implemented a home assistant by extending the state-of-the-art AM4I architecture, namely, by adding support to adaptation of interaction according to the user and context, and by evolving on previous proof-of-concept assistants. To assess the potential of our assistant to enhance living for all in smart homes, the assistant was integrated into a real scenario, with real deployed devices, and then evaluated by several end users.

The results of the study with end users show the great potential of our assistant. Nevertheless, they also show that there is still room for improvement. Deploying demonstrators in real scenarios and performing evaluations with end users can be quite challenging. However, conducting this type of evaluation is very important, as they offer a better understanding of the end users’ perception of the systems, including what they actually value, want, and need. Therefore, the results of our study constitute relevant and valuable information for future evolutions of home assistants.

### Future Work

Based on the feedback from end users, important improvements in future iterations of the home assistant include the enhancement of its initiative to alert the home’s users when an abnormal or potentially dangerous situation is detected, relying on additional information provided by the sensors of the devices and appliances. More attention should also be paid to the adaptation of interaction, especially of the presented information, to the user and context, as well as to the responsiveness of the assistant.

Regarding the demonstrator integrating our assistant and real devices, it can be improved by adding new devices. We can also extend the information that can be obtained from each device (e.g., resource consumption information) and the control commands that can be carried out with the assistant. The demonstrator can also be improved by using the graphics output modality, already included in the implementation of the home assistant, to present temporal information (e.g., temperature and resource consumption changes over time) in the form of graphs.

## Figures and Tables

**Table 1:** Overall context of the smart home’s demonstrator, aimed at a studio apartment with four divisions and several devices.

Home Division	Devices
Kitchen	Instant tap water heater, light
Living Room	Sensor box (indoor air quality and temperature), light
Office	Light, home server
Technical Room	Heat pump, air purifier, water softener

**Table 2:** The 118 words/multi-word expressions used in the 118-word test [58,62]. The words we considered as positive, neutral, and negative, in the context of our home assistant, are indicated using green, black, and red, respectively.

Accessible	Creative	Fast	Meaningful	Slow
Advanced	Customizable	Flexible	Motivating	Sophisticated
Annoying	Cutting edge	Fragile	Not secure	Stable
Appealing	Dated	Fresh	Not valuable	Sterile
Approachable	Desirable	Friendly	Novel	Stimulating
Attractive	Difficult	Frustrating	Old	Straightforward
Boring	Disconnected	Fun	Optimistic	Stressful
Business-like	Disruptive	Gets in the way	Ordinary	Time-consuming
Busy	Distracting	Hard to use	Organized	Time-saving
Calm	Dull	Helpful	Overbearing	Too technical
Clean	Easy to use	High quality	Overwhelming	Trustworthy
Clear	Effective	Impersonal	Patronizing	Unapproachable
Collaborative	Efficient	Impressive	Personal	Unattractive
Comfortable	Effortless	Incomprehensible	Poor quality	Uncontrollable
Compatible	Empowering	Inconsistent	Powerful	Unconventional
Compelling	Energetic	Ineffective	Predictable	Understandable
Complex	Engaging	Innovative	Professional	Undesirable
Comprehensive	Entertaining	Inspiring	Relevant	Unpredictable
Confident	Enthusiastic	Integrated	Reliable	Unrefined
Confusing	Essential	Intimidating	Responsive	Usable
Connected	Exceptional	Intuitive	Rigid	Useful
Consistent	Exciting	Inviting	Satisfying	Valuable
Controllable	Expected	Irrelevant	Secure	
Convenient	Familiar	Low maintenance	Simplistic	

## Data Availability

Data are contained within the article.

## Appendix A. Pre-Questionnaire

The complete list of questions of the pre-questionnaire (excluding questions 1 and 2 corresponding to the age and gender), and associated answer options, is the following (in English):

3. Do you usually use a mobile device (e.g., smartphone, tablet)?
  (a) Yes
  (b) No
4. How many times on average do you use that/these device(s)? (only presented if the answer to question 3 is “Yes”)
  (a) 0 or 1 time per day
  (b) 2 to 5 times per day
  (c) 5 to 10 times per day
  (d) More than 10 times per day
5. Do you usually use a virtual assistant (e.g., Siri, Google Assistant, Alexa)?
  (a) Yes
  (b) No
  (c) Don’t know
6. How many times on average do you use that assistant? (only presented if the answer to question 5 is “Yes”)
  (a) 0 or 1 time per month
  (b) 1 time per week
  (c) 1 time per day
  (d) More than 1 time per day
7. Have you ever used a smart speaker with an integrated assistant (e.g., Google Home, Amazon Echo)?
  (a) Yes
  (b) No
  (c) Don’t know
8. Do you usually use an assistant to obtain information about your home and/or control your home (e.g., to consult if the lights are on/off and/or turn on/off the lights)?
  (a) Yes
  (b) No
  (c) Don’t know
9. How many times on average do you use that assistant to consult information or control your home? (only presented if the answer to question 8 is “Yes”)
  (a) 0 or 1 time per month
  (b) 1 time per week
  (c) 1 time per day
  (d) More than 1 time per day

## Appendix B. First Post-Questionnaire

The complete list of questions of the first post-questionnaire, and associated answer options, is the following (in English):

1. Which of the following options best describes the assistant?
  (a) There’s no similar solution to the same problem
  (b) It’s a major improvement over currently available solutions
  (c) It’s only a minor improvement over currently available solutions
  (d) It’s similar to existing solutions
  (e) Existing solutions are more adequate
  (f) I don’t have enough knowledge on alternative solutions to answer
2. The assistant simplifies the interaction with the multiple devices of a home.
  (a) 1—Strongly disagree
  (b) 2—Disagree
  (c) 3—Neither agree nor disagree
  (d) 4—Agree
  (e) 5—Strongly agree
3. The assistant facilitates the interaction with difficult-to-access devices.
  (a) 1—Strongly disagree
  (b) 2—Disagree
  (c) 3—Neither agree nor disagree
  (d) 4—Agree
  (e) 5—Strongly agree
4. The assistant facilitates the interaction with devices without interface.
  (a) 1—Strongly disagree
  (b) 2—Disagree
  (c) 3—Neither agree nor disagree
  (d) 4—Agree
  (e) 5—Strongly agree
5. Which assistant’s functionalities do you consider the most useful? (More than one option can be selected.)
  (a) Consultation of information on the home’s state
  (b) Control of the home’s devices
  (c) Automatic alerts in abnormal or potentially dangerous situations
  (d) Integrated view of all home’s devices in a single application
  (e) Other (if this option is chosen, one or more functionalities need to be described)
6. The assistant would be easy to use by everyone.
  (a) 1—Strongly disagree
  (b) 2—Disagree
  (c) 3—Neither agree nor disagree
  (d) 4—Agree
  (e) 5—Strongly agree
7. I would be interested in using the assistant to interact with my home.
  (a) 1—Strongly disagree
  (b) 2—Disagree
  (c) 3—Neither agree nor disagree
  (d) 4—Agree
  (e) 5—Strongly agree
8. How often do you think you would use the assistant?
  (a) Multiple times a day
  (b) Once a day
  (c) A few times a week
  (d) A few times a month
  (e) A few times a year
9. Would you pay for this assistant?
  (a) Yes
  (b) No
  (c) Maybe
10. How would you prefer to acquire the assistant?
  (a) Acquisition of a device (e.g., tablet) with the assistant already installed
  (b) Rental of a device (e.g., tablet) with the assistant already installed
  (c) Acquisition of the assistant only, through a one-time purchase
  (d) Acquisition of the assistant only, through an annual subscription
  (e) Acquisition of the assistant only, through a monthly subscription
11. How much would you be willing to pay?
  (a) Less than 10 €/Less than 5 € (for monthly subscription only)
  (b) Between 10 and 100 €/Between 5 and 10 € (for monthly subscription only)
  (c) Between 100 and 1000 €/Between 10 and 50 € (for monthly subscription only)
  (d) More than 1000 €/More than 50 € (for monthly subscription only)
12. How likely are you to recommend this assistant to friends, family, or colleagues?
  (a) 1—Very unlikely
  (b) 2—Not likely
  (c) 3—Neutral
  (d) 4—Likely
  (e) 5—Very likely
13. Overall, how would you rate the assistant?
  (a) 1—Very poor
  (b) 2—Poor
  (c) 3—Fair
  (d) 4—Good
  (e) 5—Excellent
